# Simultaneous Dual Coronary Fistulas

**DOI:** 10.5935/abc.20180057

**Published:** 2018-04

**Authors:** Ioannis Ntalas, John B. Chambers, Júlia Karády, Ronak Rajani

**Affiliations:** 1 Department of Cardiology - St Thomas’ Hospital - Guy’s and St Thomas’ NHS Foundation Trust, London, United Kingdom; 2 MTA-SE Cardiovascular Imaging Research Group - Heart and Vascular Center - Semmelweis University, Budapest, Hungary

**Keywords:** Systolic Murmurs, Echocardiography, Computed Tomography Angiography

**Figure 1:**
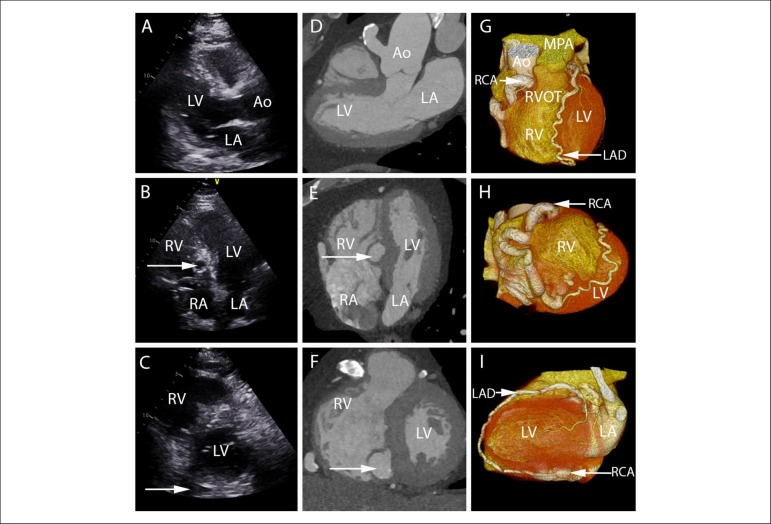
A-C) show the parasternal long axis (1A), apical 4-chamber (1B) and short axis (1C) TTE views of the left ventricle. The white arrow shows the presence of a spherical structure in the 4-chamber view and a dilated blood vessel in the short axis view. D-F) show the corresponding CTCA appearances of these findings in the same “echocardiographic views”. G) shows the 3D volume rendered image of the heart with dilated and tortuous RCA and LAD. H) shows the anatomical connection of the RCA fistula to the base of the inferior RV and a continuation of the PDA to the LAD and I) shows the LAD to PDA continuation. LV: left ventricle; RV: right ventricle; LA: left atrium; RA: right atrium; Ao: aorta; MPA: main pulmonary artery; RVOT: right ventricular outflow tract; TTE: transthoracic echocardiogram; CTCA: cardiac computed tomography angiography; RCA: right coronary artery; LAD: left anterior descending artery; PDA: posterior descending artery.

A 61-year-old man with type II diabetes mellitus was referred with breathlessness on exertion. On auscultation, there was a continuous ejection systolic murmur on the left upper sternal border. Transthoracic echocardiography showed a dilated vessel in aortic wall in the parasternal long axis view (Figure 1A) and a spherical lesion in the apical 4-chamber view (Figure 1B). A coronary computed tomographic angiographic (CTCA) study revealed a dilated and ectatic right coronary artery (RCA). It arose from the ascending aorta at the 12 o'clock position and followed a tortuous course around the right sided atrioventricular groove before passing into the basal inferoseptum draining into the base of the right ventricle. An additional fistulous connection could be detected between the posterior descending artery of the RCA and the left anterior descending artery (LAD-RCA fistula) (Figure 1G, H, I). After a normal dobutamine stress echocardiogram, a decision for continued medical therapy was taken.

Primary coronary artery fistulas (CAF) are rare congenital communications between one or more coronary arteries and a cardiac chamber or a great vessel. The RCA represents the most frequent site of origin of CAF in 60% of cases followed by the left coronary artery in 35% while two CAF are uncommon (< 5%).

The current case demonstrates the utility of CTCA in elucidating otherwise unusual transthoracic echocardiographic appearances.

